# The in vitro effects of a novel estradiol analog on cell proliferation and morphology in human epithelial cervical carcinoma

**DOI:** 10.1186/s11658-018-0079-z

**Published:** 2018-03-20

**Authors:** Laura Susan Boyd, Devrim Gozuacik, Anna Margaretha Joubert

**Affiliations:** 1 Department of Physiology, Faculty of Health Sciences, University of Pretoria, Private Bag X323, Arcadia 0007, Pretoria, South Africa; 2 Faculty of Engineering and Natural Sciences, Molecular Biology Genetics and Bioengineering Program, Sabanci University, Orhanli-Tuzla 3495, Istanbul, Turkey

**Keywords:** Estradiol analog, ESE-16, Antiproliferative, Metaphase block, Cervical carcinoma

## Abstract

**Background:**

The majority of novel chemotherapeutics target the cell cycle, aiming to effect arrest and cause apoptosis. One such agent, 2-methoxyestradiol (2ME), has been shown to possess anticancer properties against numerous cancer types, both in vitro and in vivo. Despite its promise, 2ME has exhibited limitations, including low oral bioavailability and rapid hepatic enzymatic inactivation in vivo. A novel sulphamoylated estrogen analog, 2-ethyl-3-*O*-sulphamoyl-estra-1,3,5(10)16-tetraene (ESE-16), was in silico-designed in our laboratory to overcome these issues. It was then synthesized by a pharmaceutical company and used in an in vitro antiproliferative effect study on a human cervical carcinoma (HeLa) cell line.

**Results:**

Cell proliferation data obtained from the crystal violet assay and real-time cell analysis demonstrated that 0.2 μM of ESE-16 had a significant inhibitory effect on the HeLa cells 24 h post-exposure. Immunofluorescence showed that ESE-16 is a microtubule disruptor that causes cells to undergo a mitotic block. Qualitative morphological studies using polarization-optical transmitted light differential interference contrast (PlasDIC) and light microscopy revealed a decrease in cell density and an increase in the number of cells arrested in metaphase. After ESE-16 exposure, hallmarks of apoptosis were also observed, including membrane blebbing, chromatin condensation and the presence of apoptotic bodies. Flow cytometry provided quantitative results from cell cycle progression analysis, indicating cells undergoing apoptosis and cells in the G_2_/M phase of the cell cycle, confirming cell cycle arrest in metaphase after ESE-16 treatment. Quantification of the ESE-16-mediated upregulation of cyclin B in HeLa cells and spectrophotometric and flow cytometric confirmation of cell death via apoptosis further confirmed the substance’s impact.

**Conclusion:**

ESE-16 exerts its antiproliferative effects through microtubule disruption, which induces a mitotic block culminating in apoptosis. This research provided information on ESE-16 as a potential antitumor agent and on cellular targets that could aid in the design of prospective microtubule-disrupting compounds. Further in vitro and in vivo investigations of this novel compound are needed.

## Background

Cervical carcinoma is one of the most common types of cancers in wormen, contributing 7.9% of all new cancer cases globally, which makes it the fourth most prevalent malignant disease affecting women [1]. Although there has been a decline in international incidence over the past decade thanks to increased screening and the introduction of the human papillomavirus (HPV) vaccine by the World Health Organisation in 2009, cervical cancer remains a significant disease, particularly in developing nations [1–3]. Almost 85% of new international cases occur in less developed regions, with diagnoses not taking place until the third or fourth stage of malignancy, where late diagnoses severely impacts the chances of successful treatment. Nine out of ten cervical cancer deaths occur in these areas [2–4]. The Southern African region is one of the areas categorized as high risk, with 31.5 women in every 100,000 affected [1]. This worldwide imbalance in the disease burden is probably explainable by the limited health resources in developing countries, therefore the opportunity to improve the treatment of this pathology exists, particularly at advanced stages of malignancy [4, 5].

Current treatment options include surgery, radiation therapy and chemotherapy [6]. The type of treatment selected is usually specific to the type and stage of the cancer, and the overall health of the patient [6, 7]. Chemotherapy is effective because the tumors consist of rapidly dividing cancer cells, which are more sensitive to the drugs used in this therapy [4].

Most current chemotherapeutics include anticancer drugs that target the cell cycle, aiming to arrest cancer cells in mitosis, inhibiting hyperproliferation and, eventually, leading to programmed cell death [8–11]. The compounds that particularly target microtubules and inhibit the functioning of the mitotic spindle have been established to be one of the most successful classes of chemotherapeutics to date [8, 11–14]. Microtubule-targeting agents (MTAs) disrupt the polymerization dynamics of the spindle microtubule through assorted modes, so cells are unable to pass through the spindle checkpoint to initiate anaphase, resulting in a mitosis block in metaphase [9, 15, 16]. Continual arrest in mitosis by prolonged treatment eventually causes an aberrant exit from mitosis and the cells undergo cell death, with apoptotic and autophagic characteristics having been exhibited [8, 11, 17].

2-methoxyestradiol (2ME) is an endogenous, 17β-estradiol metabolite, the antimitotic activity of which has been well documented both in vitro and in vivo. It inhibits cancer cell proliferation by targeting the microtubule network, but spares normal quiescent cells, with the effects occurring independent of estrogen receptor status [18–23]. Patented as Panzem by Entremed Inc., 2ME is currently in phase I and II clinical trials for the treatment of multiple myeloma, glioblastoma, metastatic breast-, prostate- and ovarian cancer, and a variety of other solid malignancies [24–29].

Despite its promise, 2ME has been reported to have limited bioavailability, most likely due to poor solubility, and rapid hepatic metabolism due to conjugation and oxidation of the hydroxyl groups (-OH) at C3 and C17 [23, 25, 26, 30, 31]. Researchers are attempting to exploit the positive properties of 2ME to develop novel therapeutic analogs with improved potency, increased bioavailability and slower hepatic metabolism, while maintaining its low toxicity.

Sulphamoylated derivatives of 2ME are well researched and more potent than 2ME. Their increased bioavailability and resistance to metabolism is most likely attributed to their interaction with carbonic anhydrase II (CAII) in erythrocytes, which facilitates avoidance of the first liver pass metabolism [30, 32–35]. Computational docking studies on oestrone-3-*O*-sulphamate (EMATE) revealed the compound’s ability to interact with and bind to CAII [36]. In vivo pharmokinetic findings of an EMATE derivative, 2-methoxy-*bis*-sulphamate (2-MeO2*bis*mate), in rats indicated an increased bioavailablity when compared to 2ME after oral administration [37]. Both papers reasoned that this was due to the compounds’ capability to reversibly bind to CAII in erythrocytes, thus avoiding rapid inactivation in vivo. The ability to conjugate with CAII provided the possibility for the design of CAIX inhibitors that also have the capability to disrupt mitotic microtubules. Chemical modifications to the structure of the parental 2ME (i.e., an ethyl group at C2 and a sulphamate group at position 3) improved the antiproliferative effects in nanomolar concentrations [30, 31, 38].

Taking these properties into account, novel estradiol derivatives were in silico-designed using bioinformatics software. In our lab, we demonstrated that one such analog, 2-ethyl-3-*O*-sulphamoyl-estra-1,3,5(10)16-tetraene (ESE-16), has an antiproliferative effect on various cancer cell lines with increased potency [30, 33, 39–41]. Using docking methodologies and computational analysis, Stander et al. [30] showed ESE-16’s binding ability to the active sites of CAII and CAIX. A follow-up study using gas inlet mass spectrophtometry to verify the inhibition constants (Ki) of ESE-16 on CAII and a CAIX mimic revealed that ESE-16 inhibited both CAIX and CAII, with the compound inhibiting the CAIX mimic at a lower concentration (Ki = 453 ± 43 nm) in comparison to CAII (Ki = 569 ± 61 nm). This confirmed the previous prediction from the docking studies [30, 42]. ESE-16 was therefore one of the novel analogs of 2ME synthesized for further investigation by our group.

In this study, the antiproliferative effects of ESE-16 in an epithelial cervical carcinoma (HeLa cells) are reported. This may lead to the development of a novel chemotherapeutic agent or help to progress future drugs with increased specificity and enhanced bioavailability. The research was approved by the Faculty of Health Sciences’ Ethics Committee, University of Pretoria, South Africa (L.S. Boyd, protocol number: 290/2013).

## Methods

### Cell line

HeLa cells are an immortalized, adherent, keratin-positive human epithelial carcinoma cell line derived from adenocarcinoma metastatic sites in the cervix. The cervical carcinoma cells were ATCC CCL-2 from the American Tissue Culture Collection (https://www.lgcstandards-atcc.org/).

### Compound design and synthesis

The non-commercial compound, 2-ethyl-3-*O*-sulphamoyl-estra-1,3,5(10)16-tetraene (ESE-16), was in silico-designed at the Bioinformatics and Computational Biology Unit of the University of Pretoria. Structure preparation and compound visualization were done using the Chimera package from the Resource for Biocomputing, Visualization and Informatics at the University of California, San Francisco (supported by NIH P41 RR-01081) [30]. Docking studies were performed with Autodock 4.0 and AutoDockTools4 (Scripps Research Institute) [30]. The ESE-16 compound was synthesized by iThemba Pharmaceuticals for research purposes.

### Laboratory and experimental reagents

All experimental chemicals were of cell culture analytical grade and purchased from Sigma Aldrich, unless otherwise specified. Dulbecco’s modified Eagle’s medium (DMEM), heat-activated fetal calf serum (FCS) and sterile culture flasks were purchased from Separations. Streptomycin, penicillin and fungizone were supplied by Highveld Biological. Hematoxylin, eosin, ethanol and xylol were purchased from Merck Chemicals. The xCELLigence E-Plates were acquired from Anatech Instruments. The anti-cyclin B1-fluorescein isothiocyanate-conjugated (FITC-conjugated) primary antibody was purchased from Millipore Corporation and the Annexin V-FITC Apoptosis detection and Caspase 3 Colorimetric kits were obtained from BioVision. Alpha-tubulin antibody, alexafluor-488 and 4′,6-diamidino-2-phenylindole (DAPI) were supplied by BIOCOM Biotech.

### Cell culture

HeLa cells were grown as monolayers in either 25- or 75-cm^2^ sterile culture flasks in DMEM containing sodium pyruvate, glucose and L-glutamine supplemented with 10% filtered FCS, 100 μg/l streptomycin, 100 μg/l penicillin and 250 μg/l fungizone. Cells were maintained at 37 °C in a humidified environment, containing 5% CO_2_ in a Forma water-jacketed incubator (Thermo Fischer Scientific).

### General experimental procedures

Experiments were performed in either 96-well tissue culture plates (5 × 10^3^ cells per well in 200 μl of medium), 6-well plates on heat-sterilized coverslips (25 × 10^4^ cells per well in 3 ml of medium) or in 25-cm^2^ culture flasks (5 × 10^5^ cells per flask in 4 ml of medium). Prior to exposure to the relevant concentration(s) of the compound, the cells were seeded and incubated at 37 °C in 5% CO_2_ for 24 h to allow for attachment and then provided with fresh growth medium.

Stock solutions of ESE-16 were dissolved in dimethyl sulphoxide (DMSO) to a concentration of 1 mM (stored at − 20 °C) and diluted with growth medium to the desired concentration of ESE-16 prior to exposure. Appropriate controls were included in all experimentation: cells propagated in medium only and a vehicle control where cells were treated with DMSO in combination with growth medium with the final dilutions never exceeding 0.05% (*v*/v). Exposure to actinomycin D (0.1 μg/ml in growth medium) was used as a positive control for apoptosis.

### Cell proliferation

#### Crystal violet staining

Spectrophotometry using crystal violet staining was employed to quantify the number of fixed monolayer cells. The triphenylmethane dye stains the DNA of the cell nucleus [43]. Cell number was determined by solubilising the adsorbed dye in a triton X-100 solution and quantifying the optical density of the solution. The optical density or absorbance measured is directly related to the number of viable cells [43, 44].

HeLa cells were seeded in 96-well tissue culture plates in complete growth medium and incubated for 24 h at 37 °C to allow for attachment. The cells were exposed to a dilution series of ESE-16 ranging from 0.1–0.3 μM for 24, 48 and 72 h. After each exposure period, the experiment was terminated by removal of the media and fixation of the cells with 100 μl of 1% glutaraldehyde. After 15 min of incubation at room temperature, the glutaraldehyde was discarded and the cells were stained with 100 μl of 0.1% crystal violet stain for a further 30 min at room temperature. The plates were then submersed under running tap water to remove excess stain and left to dry overnight. The dye was solubilized using 200 μl of 0.2% triton X-100. After 30 min incubation at room temperature, 100 μl of the solution was transferred to a clean 96-well plate and the absorbance was determined at 570 nm using an ELX800 Universal Microplate Reader (BioTek Instruments Inc., Analytical Diagnostic Products).

#### xCELLigence real time cell analysis

The xCELLigence system was developed by Roche Applied Sciences and ACEA Biosciences. It provides an accurate platform to non-invasively quantify and gather information on adherent cell proliferation, viability and cytotoxicity in real-time in a label-independent manner [45, 46]. The system uses an electronic readout called impedance generated by adherent cell interaction with gold microelectrodes integrated on the bottom of each well of the 16- or 96-well microtiter plates [45, 46]. A change in impedance is expressed as a parameter termed the cell index (CI) that measures the general status of the cells. Detachment due to cell death results in a decrease in electrical impedance and thus in CI [46]. An accurate analysis profile of the HeLa cells in response to ESE-16 exposure was generated by plotting the CI values over time.

An initial titration was performed prior to experimentation to determine the experimental seeding density that would produce the optimal cell index at the reference time. It was determined to be 5 × 10^3^ cells per 250 μl.

For experimentation, 100 μl of growth medium was added for background quantification. Subsequently, 5 × 10^3^ cells were seeded per well and allowed to settle in a laminar flow cabinet for 30 min prior to being placed in the xCELLigence system housed in a Forma water-jacketed incubator (37 °C, 5% CO_2_) [47]. Following cell attachment (24 h), 50 μl of a ESE-16 concentration series ranging from 0.1–0.5 μM and the appropriate controls were added, giving to a final volume of 250 μl per well. The E-Plates were then monitored continuously for 96 h. The results obtained from the xCELLigence system were analyzed using RTCA Software version 1.2.1 (ACEA Biosciences).

### Cell morphology

#### Polarization-optical transmitted light differential interference contrast microscopy

Unlike conventional differential interference contrast, polarization-optical transmitted light differential interference contrast (PlasDIC) linear polarized light is generated behind the objective, producing high-quality three-dimensional images of individual cells, clusters of cells, and thick individual cells (such as metaphase blocked cells) in plastic culture dishes [48, 49]. This technique was used to qualitatively evaluate the effects of ESE-16 on HeLa cell morphological characteristics, and to view the cells in mitotic arrest and/or undergoing cell death.

Cells were seeded appropriately for the specific experimentation in a suitable culture vessel. After 24 h allowance for cell attachment, cells were photographed pre- and post-treatment with the appropriate controls (*n* = 3). Images were captured using the Zeiss Axiovert-40 microscope (Carl Zeiss AG).

#### Light microscopy: Hematoxylin and eosin staining

To observe the influence of ESE-16 on morphological changes in the HeLa cells, hematoxylin and eosin (H&E) staining was used, providing both qualitative and quantitative (mitotic indices) data. H&E stain the nucleus blue-purple and the cytoplasm pinkish-red, allowing identification of the different mitotic phases of the cell, including any hallmarks of apoptosis [50, 51].

Following seeding on heat-sterilized coverslips and 24 h for attachment, cells were exposed to ESE-16. At termination, the coverslips were transferred to staining dishes and fixed with Bouin’s fixative for 30 min followed by 70% ethanol exposure at room temperature for 20 min. Then the slides were rinsed with tap water. Mayer’s hematoxylin was added for 20 min, and the coverslips were rinsed in running tap water for 2 min and then rinsed with 70% ethanol. The cells where stained with eosin (1%) for 5 min before dehydration and consecutive rinsing with 70, 96 and 100% ethanol and xylol twice for 5 min. The stained coverslips were then mounted onto microscope slides with resin (Entellan) and allowed to dry (24 h) prior to evaluation using a Zeiss Axiovert MRs microscope (Carl Zeiss AG). Mitotic indices were determined by counting 1000 cells on each slide of the replicates and expressing the percentage of cells in each phase of mitosis and interphase, as well as abnormal and/or apoptotic cells.

#### Confocal microscopy

Microtubules are composed of α- and β-tubulin heterodimers, the polymerization dynamics of which are essential for cell progression through mitosis. They are often targeted with chemotherapeutic agents to induce mitotic arrest. Confocal microscopy using immunofluorescence was used to determine whether ESE-16, like its parental molecule, induces morphological changes in the microtubule network of actively dividing HeLa cells [30, 48].

Exponentially growing HeLa cells (250,000) were seeded onto sterile coverslips in 6-well culture plates and allowed to undergo attachment. Subsequently, cells were exposed to ESE-16 for 24 h. To terminate the experiment, the cells were fixated with a glutaraldehyde fixer (0.3%) for 10 min at 37 °C and then rinsed three times with cytoskeletal buffer followed by permeabilization (1% triton X-100 in cytoskeletal buffer) for 15 min at room temperature. Further rinsing was as follows: with the cytoskeletal buffer for 3 min and twice with phosphate-buffered saline (PBS) for 5 min between consecutive 60 min incubations at 37 °C with 100 μl of mouse monoclonal α-tubulin antibody followed by anti-mouse IgG 58 FITC-conjugated secondary antibody was performed. After washing three times with PBS for 5 min each, the coverslips were counterstained with DAPI as a blue nuclear stain for 7 min. The stained coverslips were washed again with PBS before being mounted with a glycerol-based mounting fluid and viewed with a Zeiss LSM 510 META confocal laser microscope (Carl Zeiss AG) at the Laboratory for Microscopy and Microanalysis at the University of Pretoria.

### Flow cytometry

#### Cell cycle progression

Cellular DNA content was analyzed via flow cytometry to provide information on the distribution of cells in the various stages of the cell cycle after ESE-16 exposure. Propidium iodide (PI) is a fluorogenic, nuclear stain that stoichiometrically binds to nucleic acids, with fluorescence emissions directly associated to the DNA content of the cell. Therefore, the subpopulations of cells can be differentiated via fluorescence intensity due to cells having varying amounts of DNA in the assorted stages of the cell cycle [52, 53]. As PI binds to DNA, it also reacts with double-stranded RNA. RNase A was therefore added to the staining solution to digest any RNA prior to flow cytometry [54]. The phases identified were G_1_, S (DNA synthesis) and G_2_/M (mitotic). Cells in the sub-G_1_ (apoptotic population) category were also detected due to their low DNA content because of nuclear fragmentation [52].

After seeding in 25-cm^2^ culture flasks and allowing for cell attachment overnight, HeLa cells were exposed to 0.2 μM ESE-16 for 24 h. Following trypsinization, the cells were resuspended in 1 ml growth medium. They were then pelleted by centrifugation (300 x *g*) for 5 min and resuspended in 1 ml ice cold PBS. The cells were further washed and resuspended in 200 μl of ice-cold PBS containing 0.1% FCS. Ice-cold 70% ethanol (4 ml) was added in a drop wise manner and stored at 4 °C for 24 h. The following day, the cells were pelleted, the supernatant was discarded and the cells were resuspended for incubation in 1 ml of PBS containing PI (40 μg/ml), RNAse A (100 μg/ml) and triton X-100 (0.1%) at 37 °C, 5% CO_2_ for 45 min. PI fluorescence was measured using a fluorescence activated cell sorting (FACS) FC500 System flow cytometer, equipped with an air-cooled argon laser (Beckman Coulter) with at least 30,000 cells per sample evaluated using CXP software (Beckman Coulter). The data from cell debris and aggregated cells were excluded from analyses. Cyflogic 1.2.1 (CyFlo) software was used to calculate cell cycle distributions by assigning relative DNA content per cell to sub-G_1_, G_1_, S and G_2_/M fractions.

#### Cyclin B detection

As a regulatory subunit of cyclin-dependant kinase (CDK) 1, cyclin B is essential for mitotic initiation. The cyclin B-CDK1 complex is necessary for the G_2_/M-phase transition of the cell cycle, so any abnormalities in spindle formation or misalignment of spindle–kinetochore connections initiate the spindle checkpoint to inhibit cyclin B degradation [55]. An increased level of this substrate would indicate a metaphase arrest [56, 57]. A cyclin B1–FITC-conjugated antibody was used to flow cytometrically quantify the presence of the cyclin B1 protein and confirm whether ESE-16 exposure induces HeLa cells to undergo mitotic arrest.

HeLa cells were seeded in 25-cm^2^ culture flasks. Following exposure to ESE-16, the cells were trypsinized and washed with 1 ml ice-cold PBS. After centrifugation (15,000 x *g*), the supernatant was discarded and the cells were resuspended in 200 μl of ice-cold PBS containing 0.1% FCS. After fixation with 10 ml ice-cold 70% ethanol, the fixed cells were stored at 4 °C for 24 h. Next, the cells were washed twice with 500 μl PBS to remove the excess ethanol. The antibody solution was prepared by diluting the anti-cyclin B1–FITC-conjugated primary antibody with PBS (1:5). The diluted antibody (10 μl) was added together with 90 μl PBS containing 0.1% triton X-100 (to a final volume of 100 μl). Subsequent to incubation with the primary antibody for 40 min at 37 °C (protected from light), the cells were washed twice with 1 ml PBS and resuspended in 0.6 ml PBS for analysis. FITC fluorescence was measured with a FACS FC500 System flow cytometer, equipped with an air-cooled argon laser (Beckman Coulter). At least 30,000 cells per sample were evaluated using the CXP software. Analysis of the data and histogram formation was performed using the Cyflogic 1.2.1 (CyFlo) software.

### Determination of cell death

#### Apoptosis detection based on phosphatidylserine translocation

In early apoptosis, calcium-dependent scramblase activity is activated, causing the external translocation of a membrane phospholipid called phosphatidylserine (PS) [58]. This externalization provides binding sites for annexin V, a 35- to 36-kDa, calcium-dependent phospholipid-binding protein with a high affinity for PS. Annexin V is conjugated to FITC for the quantitative detection and analysis of apoptosis. Simultaneous staining of the cells with PI allows for differentiation between necrotic and apoptotic cells. In necrotic cells, there is a complete dissolution of the plasma membrane and cell lysis, allowing PI to enter the cell and stain the nucleus [59].

Cells were seeded in a 25-cm^2^ flask, incubated for 24 h and subsequently exposed to ESE-16. After trypsinization and washing in 1 ml PBS, the cells were resuspended in 100 μl of 1× Binding Buffer. Annexin V–FITC (5 μl) was then added together with 10 μl of PI (100 μg/ml) and gently vortexed followed by incubation in the dark for 15 min at room temperature. Binding Buffer (400 μl) was added and immediately the annexin V and PI fluorescence were analyzed with a FACS FC500 System flow cytometer equipped with an air-cooled argon laser (Beckman Coulter) excited at 488 nm. Data from at least 30,000 cells were analyzed with CXP software, with data analysis performed using the Cyflogic 1.2.1 software (CyFlo).

#### Apoptosis detection based on caspase 3

The protease caspase 3 is an important executioner caspase in the apoptotic pathway, indicated in specific events, i.e., DNA degradation, membrane blebbing and nuclear condensation, all of which lead to programmed cell death [60]. Spectrophotometry was used to determine the level of caspase 3 activity to ascertain apoptosis induced by ESE-16 is caspase-dependent.

Cells (500,000) were seeded into 25-cm^2^ culture flasks. Following an overnight attachment policy, exposure of ESE-16 and trypsinization, the cells were pelleted, resuspended in 50 μl ice-cold Cell Lysis Buffer and incubated on ice for 10 min. They were then centrifuged for 1 min and the supernatant was transferred to a clean tube and put on ice. 2× Reaction Buffer (50 μl) and 5 μl of the 4 mN DEVD-*p*NA substrate were subsequently added to the supernatant and the samples then incubated for 1 h at 37 °C. The absorbance was measured at 400 nm using an EL_X_800 Universal Microplate Reader (BioTek Instruments Inc., Analytical Diagnostic Products).

### Statistical analyses

PlasDIC, light and confocal microscopy were analyzed qualitatively, with H&E staining providing both quantitative (mitotic indices) and qualitative data. The xCELLigence, flow cytometric (cell cycle progression, cyclin B1 and annexin V–FITC) and spectrophotometric (crystal violet and caspase 3 colorimetric assays) studies were analysed quantitatively. Data from the mitotic indices were obtained by counting 1000 cells on each slide of the replicates, repeated three times. Flow cytometric data for no less than 30,000 events were counted for each sample, with three independent repeats conducted. The data were analyszd with Cyflogic 1.2.1 (Perttu Terho & Cyflo Ltd). ANOVA and Students’ *t*-test were used to determine the analytical variation in experimental procedures and biological variations within each experiment. Means are represented in bar charts, with T-bars demonstrating standard deviation. A *p* value < 0.05 was regarded as statistically significant.

## Results

### Cell proliferation

#### Crystal violet staining

**Fig. 1:**
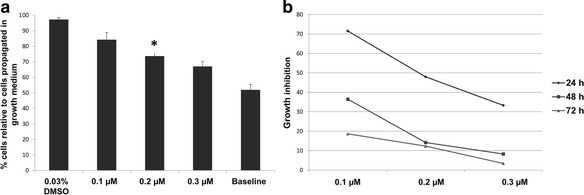
The effects of ESE-16 on HeLa cell proliferation. **a** The HeLa cell numbers are expressed as a % of cells relative to cells in growth medium only after 24 h of exposure to ESE-16 (0.1, 0.2 and 0.3 μM). Vehicle-treated control cells (0.03% DMSO) are also shown. A significant antiproliferative effect was observed after exposure to 0.2 μM ESE-16. **b** The growth inhibitory effect of ESE-16 after 24, 48 and 72 h. A growth inhibition of 48% was observed after 24 h exposure to 0.2 μM ESE-16. *p < 0.05

Dose- and time-dependent studies were done via spectrophotometry to demonstrate the effects of ESE-16 on HeLa cell proliferation. Crystal violet staining revealed a statistically significant inhibition of cell growth after exposure to 0.2 μM ESE-16 (*p* < 0.05; Fig. 1a). A growth inhibition (GI_50_) of 48% was calculated for 0.2 μM ESE-16 after a 24 h exposure period (Fig. 1b). This determined GI_50_ concentration and exposure time (24 h) was employed in all subsequent experiments.

#### xCELLigence real-time cell analysis

**Fig. 2:**
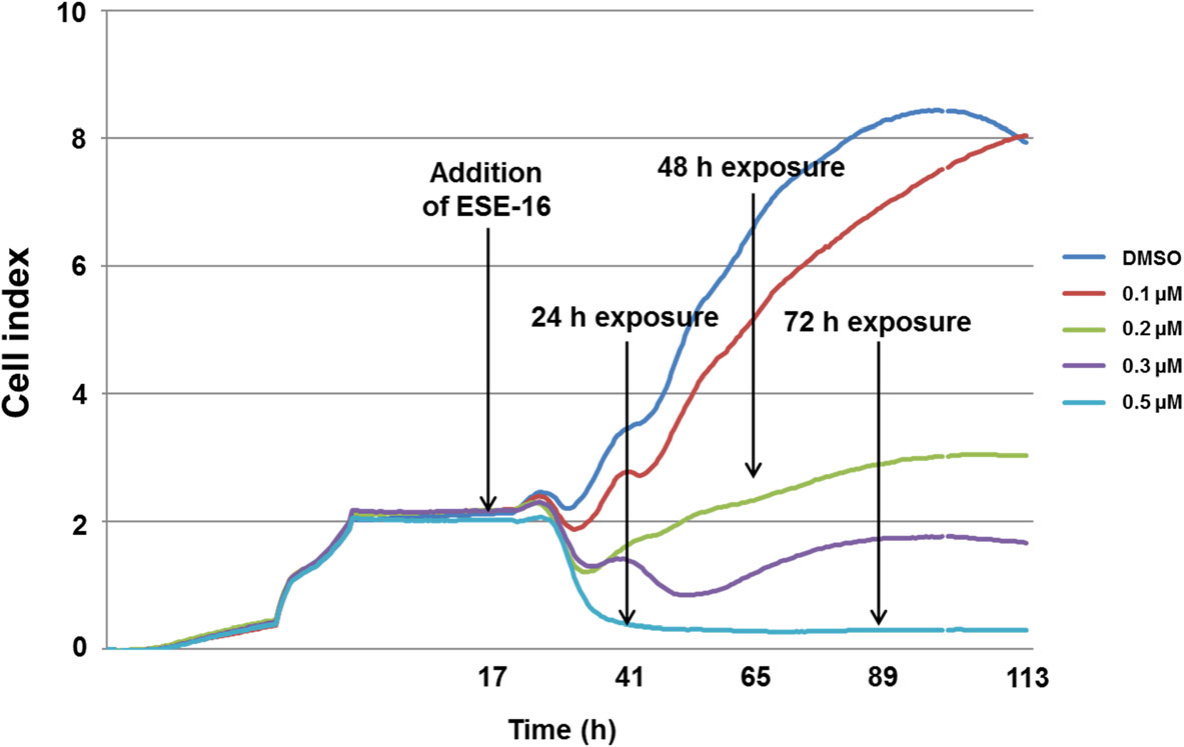
Real-time cell monitoring illustrating an analysis profile of the HeLa cells in response to ESE-16 exposure. Cell growth was significantly reduced by 0.2–0.5 μM ESE-16 after 24 h exposure

This real-time label independent technique provided the ability to quantify proliferation and adhesion characteristics of HeLa cells after 96 h continuous ESE-16 exposure. By plotting the cell index (CI) values over time using the xCELLigence RTCA software, an accurate analysis profile of the HeLa cells in response to ESE-16 exposure was generated. Each curve represents an average of three replicates. The RTCA approach revealed HeLa cell proliferation was significantly reduced by 0.2, 0.3 and 0.5 μM ESE-16: all three caused a decrease in the cell index when compared to the vehicle-treated control cells (Fig. 2).

### Cell morphology

#### Polarization-optical transmitted light differential interference contrast microscopy

**Fig. 3:**
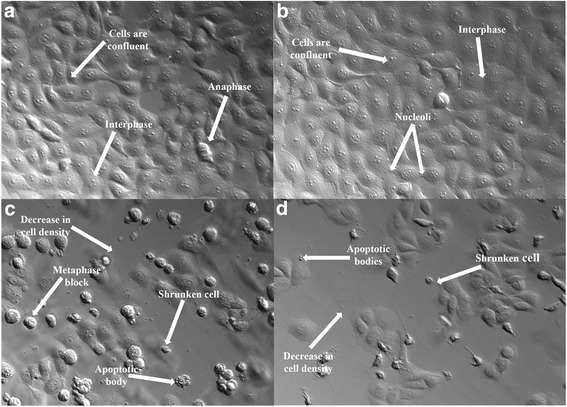
PlasDIC images of HeLa cells demonstrating the morphological changes induced by ESE-16. **a** and **b** Cells propagated in growth medium (**a**) and vehicle-treated control cells (**b**) were confluent, with majority of cells in interphase. **c** ESE-16-treated cells: several cells are blocked in metaphase and there are visible apoptotic characteristics such as shrunken cells and apoptotic bodies. **d** Hallmarks of apoptosis were observed in the positive control cells, which were exposed to actinomycin D (20× magnification)

PlasDIC images of cells were taken after 24 h exposure to visualize the in vitro effects of ESE-16 on the morphology of HeLa cells and to observe any features of cell death. There were pronounced morphological differences in ESE-16-treated cells, including compromised cell density when compared to cells propagated in medium and the vehicle-treated control cells (Fig. 3a and b). Like the cells with apoptosis induced using actinomycin D, ESE-16-treated cells also showed an increase in the number of cells present in metaphase, together with shrunken cells and apoptotic bodies, indicative of cell death via apoptosis (Fig. 3c and d).

#### Light microscopy: Hematoxylin and eosin staining

**Fig. 4:**
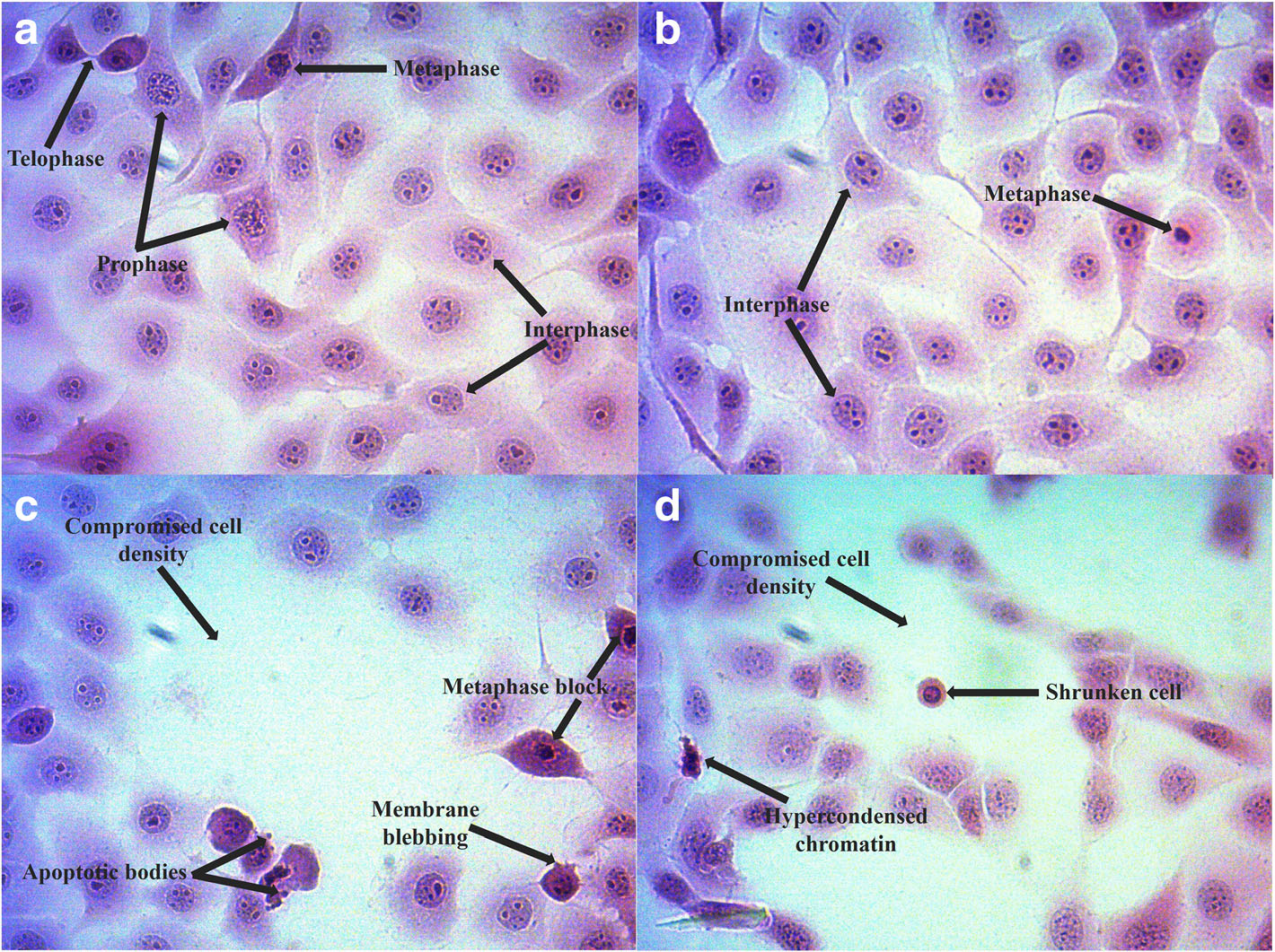
Light microscopy images revealing the morphological effects of ESE-16 on the nuclear and cytoplasmic structures in HeLa cells. **a** and **b** Cells propagated in growth medium (**a**) and vehicle-treated control cells (**b**) showed a dense population and normal division. Most cells were found to be in interphase. **c** and **d** Cells exposed to ESE-16 (**c**) and 0.1 μg/ml actinomycin D (**d**) revealed membrane blebbing, apoptotic bodies and an increased number of metaphase cells after 24 h. Both treatments resulted in a compromised cell density (40× magnification)

Hematoxylin and eosin (H&E) staining supported a qualitative analysis of the morphological effects of ESE-16 on HeLa cell nuclear and cytoplasmic machinery. Cells propagated in complete growth medium (Fig. 4a) and the vehicle-treated control cells (Fig. 4b) showed normal morphology and cell division with no signs of distress. The ESE-16 exposed cells (Fig. 4c) and the positive control (actinomycin D-treated) cells (Fig. 4d) revealed an increase in the number of metaphase cells, compromised cell density and characteristics of apoptosis, such as membrane blebbing and the presence of apoptotic bodies.

**Fig. 5:**
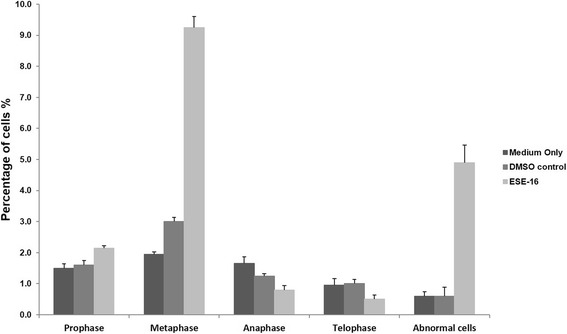
Bar graph indicating the mitotic indices of HeLa cells propagated in medium or DMSO with or without ESE-16 exposure. Cells treated with ESE-16 demonstrated increased numbers in metaphase and apoptotic hallmarks when compared to the controls

To support observations from H&E staining, mitotic indices were determined by identifying the number of cells present in interphase, mitotic phases and cells undergoing apoptosis (abnormal cells). This was achieved by counting 1000 cells on each slide of the biological replicates. Semi-quantitative data indicated an increase in the number of cells in metaphase (9.25%) and apoptotic cells (4.9%) after 24 h of exposure to ESE-16 when compared to the vehicle control (3% in metaphase, 0.6% abnormal cells; Fig. 5).

#### Confocal microscopy

**Fig. 6:**
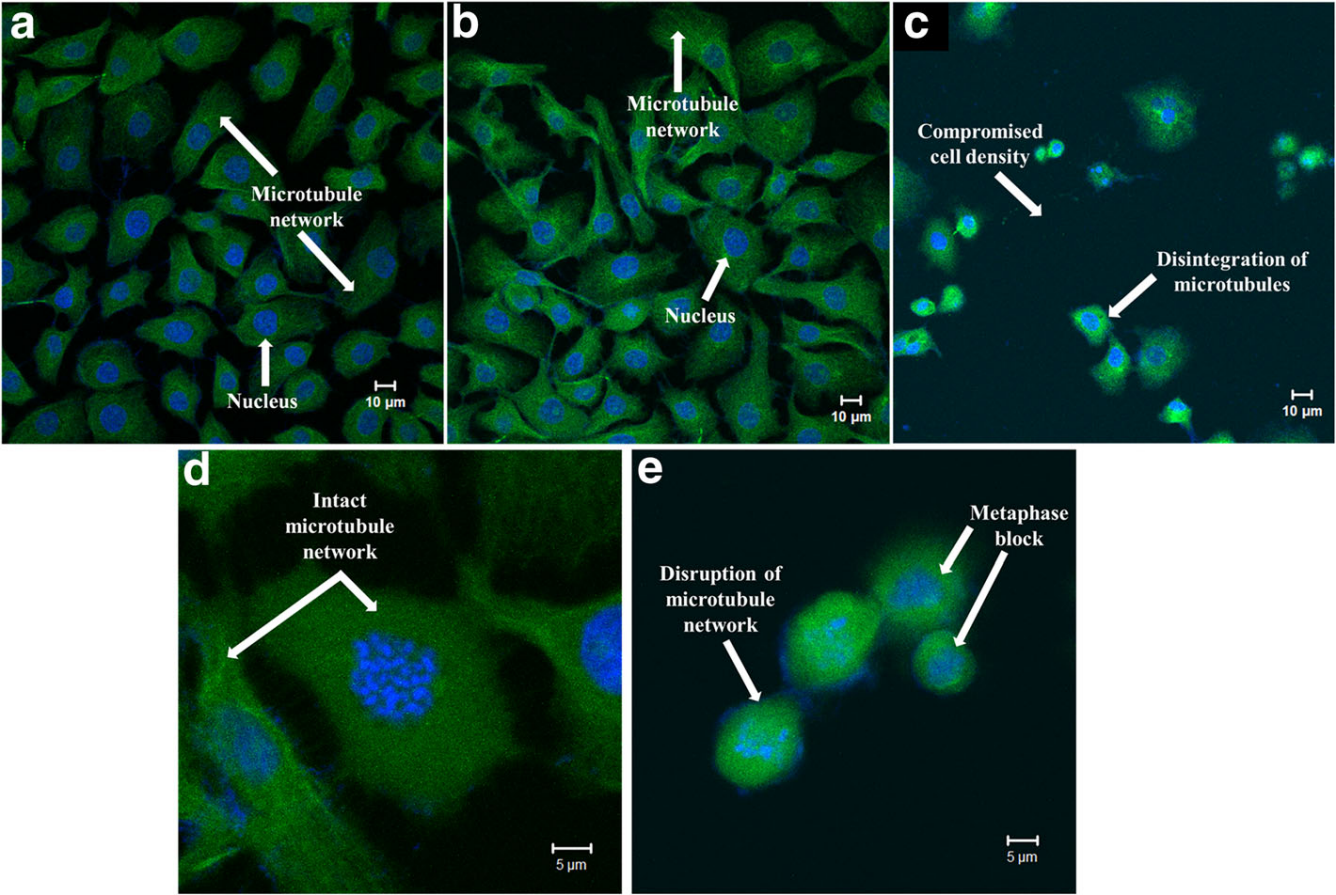
Confocal microscope images of the tubulin structure in HeLa cells using immunofluorescence staining. **a** and **b** Medium-propagated cells (**a**) and vehicle-treated control cells (**b**) showed normal and intact microtubules. **c** HeLa cells treated with ESE-16 revealed a decrease in cell density and a complete loss and disruption of the microtubule network. **d** A HeLa cell in prophase with an intact microtubule network. **e** A HeLa cell after 24 h exposure to ESE-16, revealing an increased number of cells in metaphase with disintegrated microtubules

Tubulin morphology was examined via immunofluorescence using an α-tubulin antibody and DAPI to determine the influence of ESE-16 on the microtubule network of cervical cancer cells. Intact microtubules with normal construction were observed from HeLa cells propagated in growth medium (Fig. 6a) and vehicle-treated control cells (Fig. 6b). However, ESE-16-treated cells showed decreased cell density, disruption and breakdown of the microtubule network, and an increased number of cells in metaphase (Fig. 6c).

### Flow cytometry

#### Cell cycle progression

**Fig. 7:**
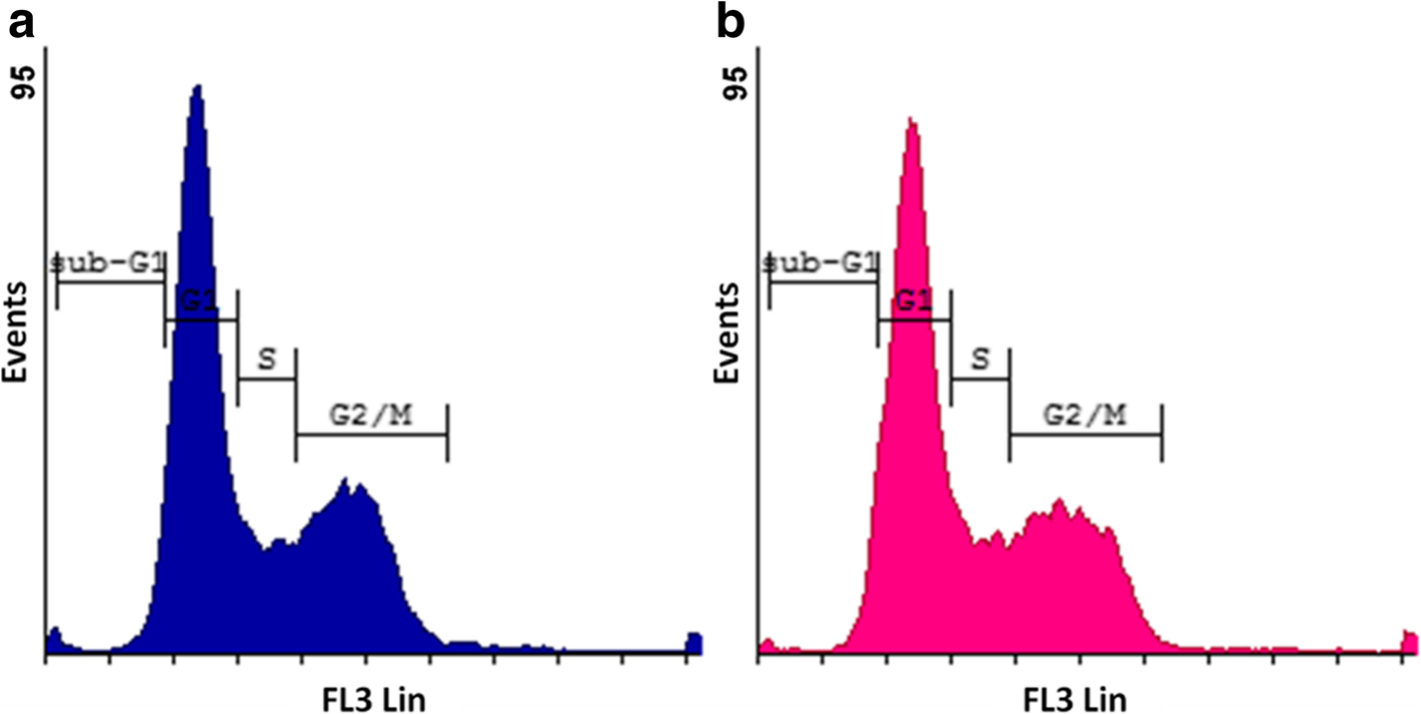
Histograms of the cell cycle distribution of HeLa cells. Cells propagated in growth medium (**a**) and vehicle-control cells (**b**) showed normal cell cycle profiles after 24 h

**Fig. 8:**
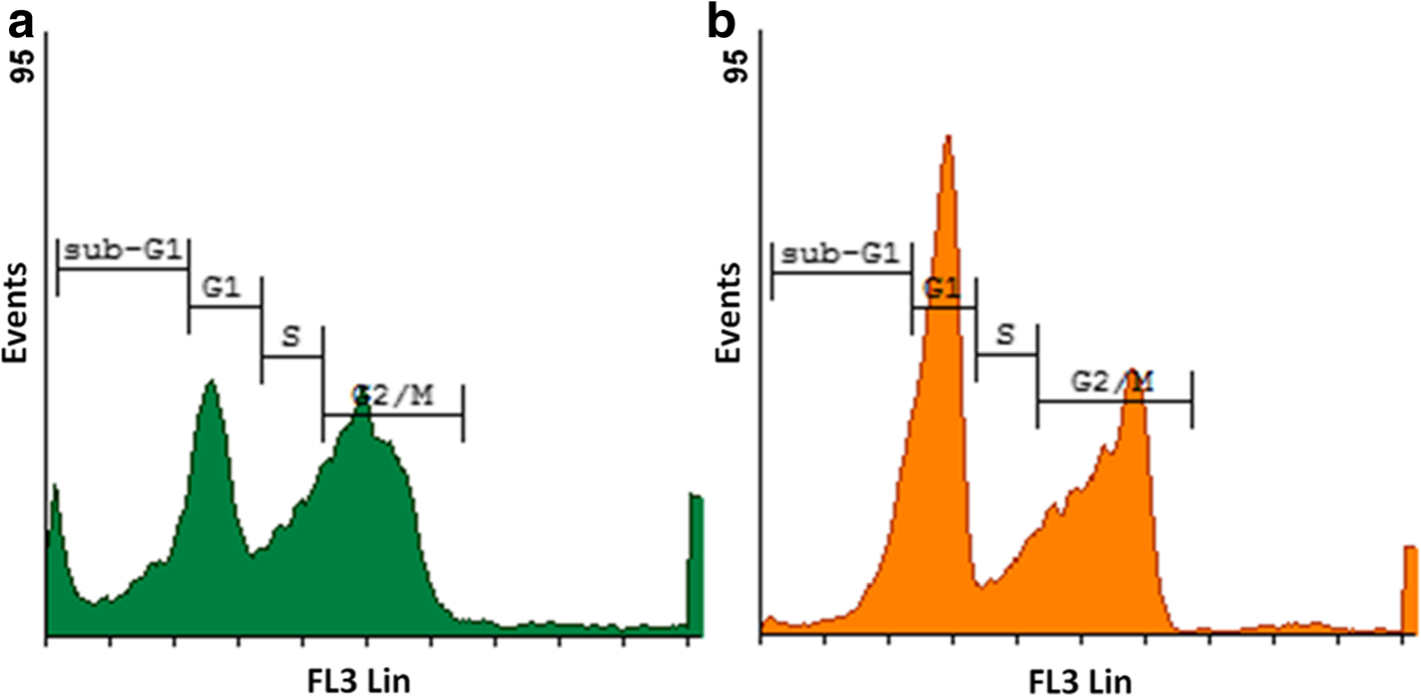
Histograms of the cell cycle distribution of HeLa cells. Cells after 24 h of exposure to 0.2 μM ESE-16 (**a**) and actinomycin D (**b**). After ESE-16 exposure, there was a significantly increased number of cells in apoptosis (sub-G_1_) and mitotic arrest (G_2_/M)

**Fig. 9:**
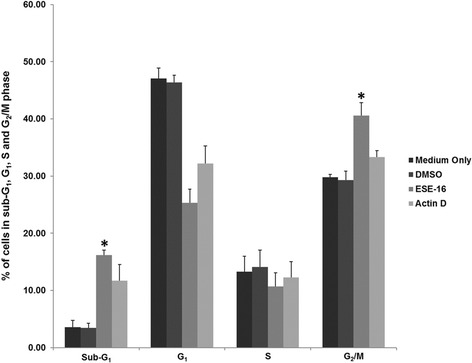
Bar graph indicating the percentage of HeLa cells in the sub-G_1_, G_1_, S and G_2_/M phases. The data revealed a statistically significant increase in cells present in the sub-G_1_ and G_2_/M phase after ESE-16 treatment when compared to controls. *p < 0.05

Cellular DNA content was analysed using PI to investigate the in vitro effects of ESE-16 on cell cycle progression. Evaluation of the cell subpopulations revealed a statistically significant number of ESE-16-treated cells in the G_2_/M phase (41%) when compared to cells propagated in medium (30%) and the vehicle-treated control cells (29%), as well as those in sub-G_1_ (16%; Figs. 7, 8 and 9). Results indicate a metaphase block (increase in G_2_/M) and the presence of apoptosis (increase in sub-G_1_), suggesting that the growth of the HeLa cells is inhibited in vitro by ESE-16 through mechanisms that induce cell cycle arrest and apoptosis.

#### Cyclin B detection

**Fig. 10:**
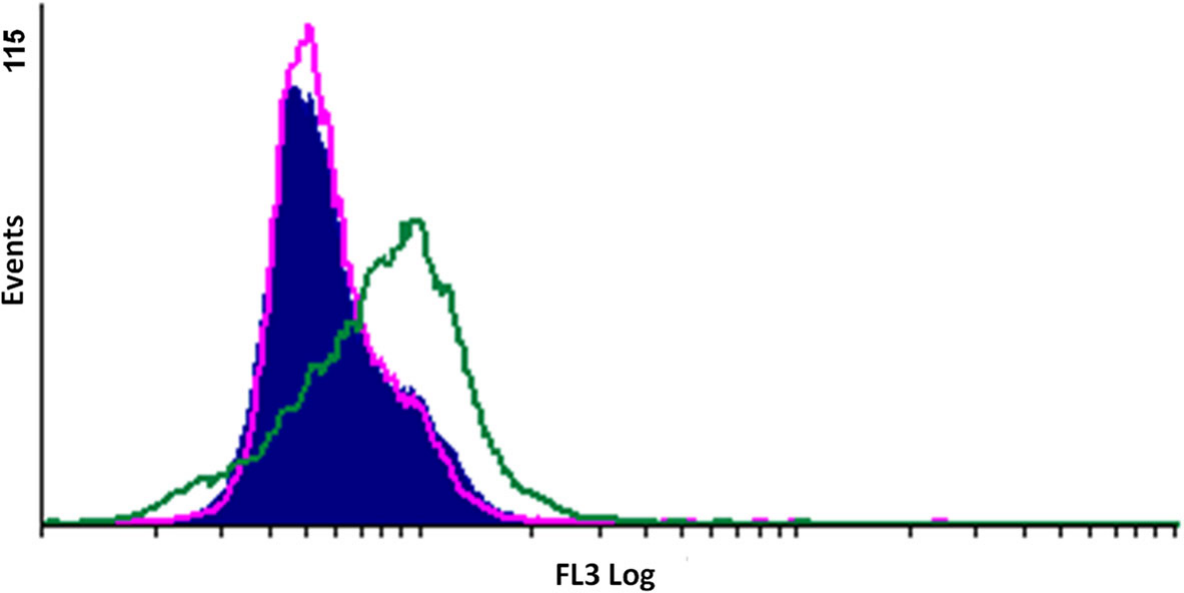
Overlay histogram for the mean fluorescence intensity of cyclin B1 expression. An increase in the upregulation of the cyclin B1 proteins was observed in HeLa cells exposed to ESE-16 (green) compared to the vehicle-control cells (pink) and cells propagated in growth medium (blue)

**Fig. 11:**
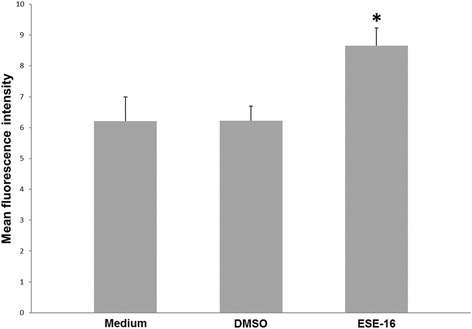
Bar graph of the average mean fluorescence intensity measured for the upregulation of cyclin B1 proteins after HeLa cell exposure to ESE-16. The increase in the MFI indicates cyclin B1 upregulation compared to cells in growth medium and the vehicle-treated control cells. *p < 0.05

The degradation of cyclin B is necessary for the cell cycle to transition through the G_2_/M phase, so an increase of cyclin B would indicate a metaphase arrest. Flow cytometry using a cyclin B1–FITC-conjugated antibody was used to quantify cyclin B1 protein upregulation and to confirm the ability of ESE-16 to induce a metaphase block in the HeLa cells. There was an overall 1.4-fold increase in cyclin B1 compared to the controls (Fig. 10). The results are the average mean fluorescence intensity (MFI) of all three repeats. The ESE-16-treated cells had a statistically significant increased MFI of 8.65 compared to the vehicle-treated control (6.22) cells and those propagated in growth medium (6.21; Fig. 11).

### Determination of cell death

#### Apoptosis detection based on phosphatidylserine translocation

The translocation of phosphatidylserine (PS) from the inner to the outer plasma membrane is considered an early sign of apoptosis [61]. The ESE-16-mediated induction of this early apoptotic event in HeLa cells was investigated using annexin V conjugated to FITC.

**Fig. 12:**
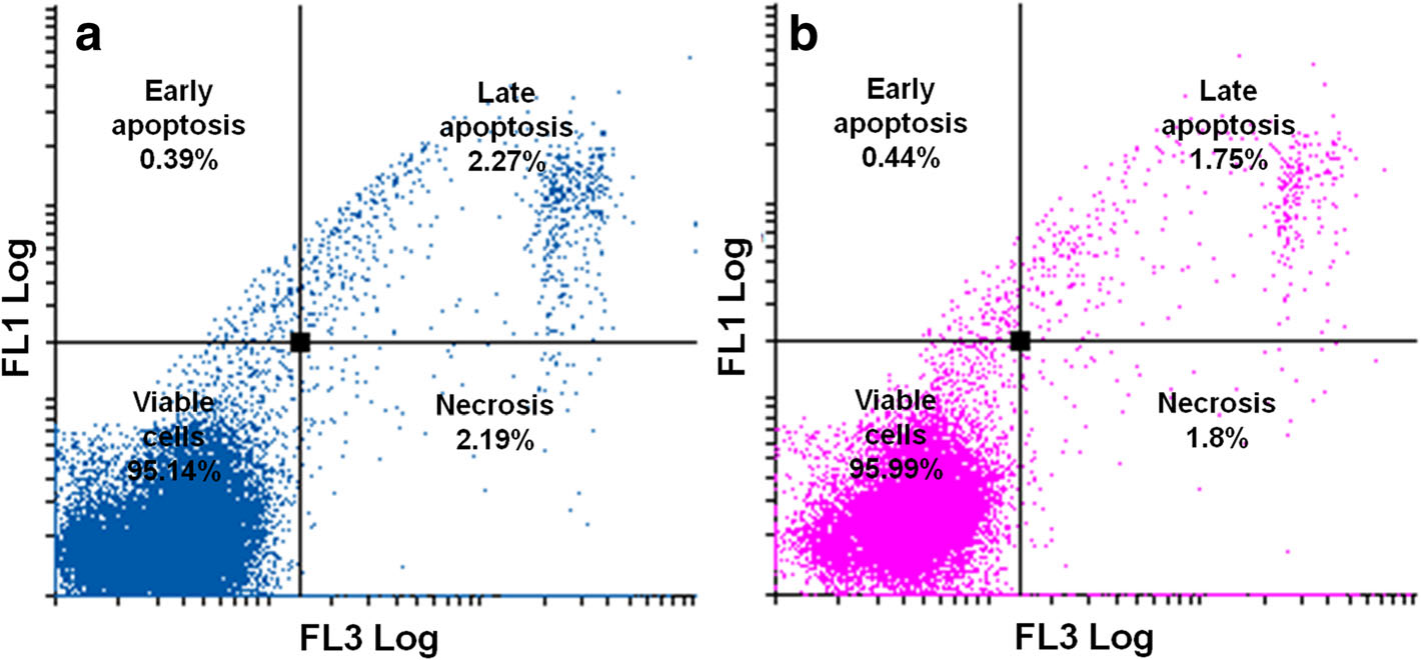
Dot plots of cells indicating the occurrence of apoptosis represented as annexin V (FL1 Log) versus PI (FL3 Log). Cells propagated in the growth medium (**a**) and vehicle-treated control cells (**b**) showed a cell viability of over 95%

**Fig. 13:**
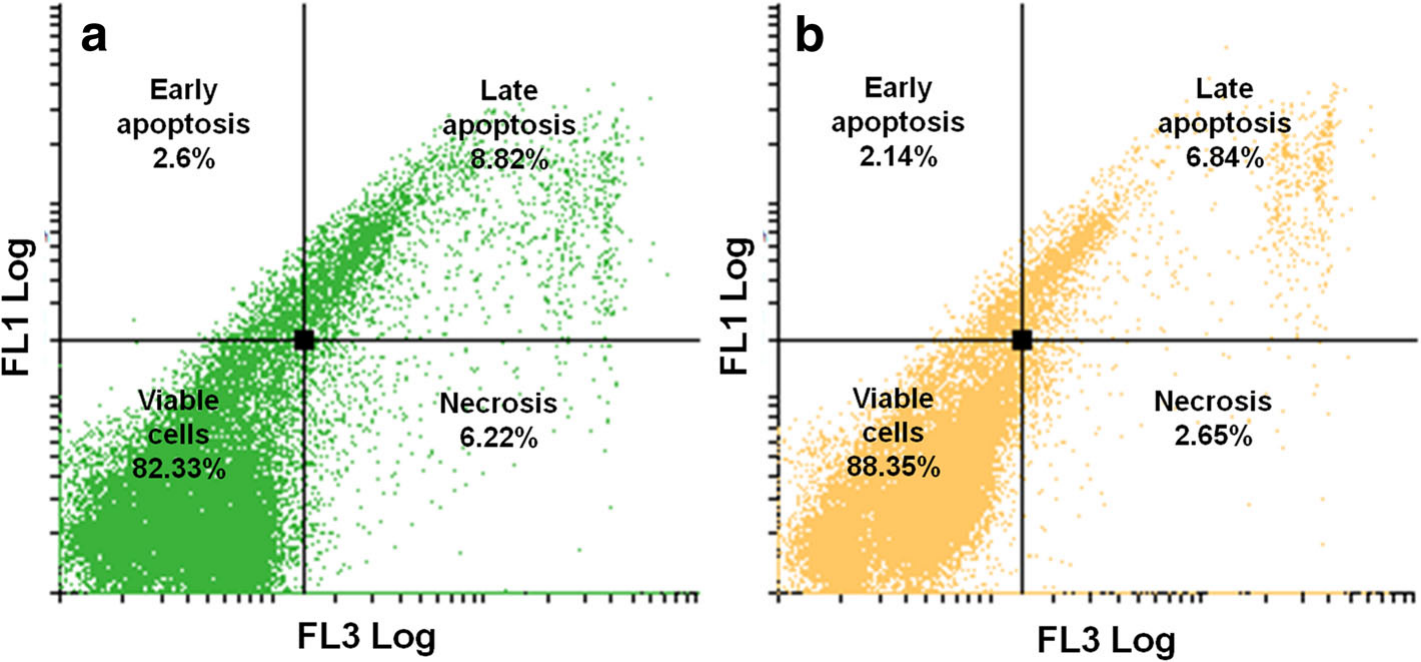
Dot plots of cells indicating the occurrence of apoptosis represented as annexin V (FL1 Log) versus PI (FL3 Log). **a** ESE-16-treated cells: 82.33% were viable, 2.6% were in early apoptosis, 8.82% were in late apoptosis and 6.22% were undergoing necrosis. **b** Actinomycin D treatment gave similar results

After exposure to ESE-16, a higher number of cells were in early (2.6%) and late apoptosis (8.82%) when compared to the cells propagated in growth medium and vehicle-treated control cells (Figs. 12 and 13).

**Fig. 14:**
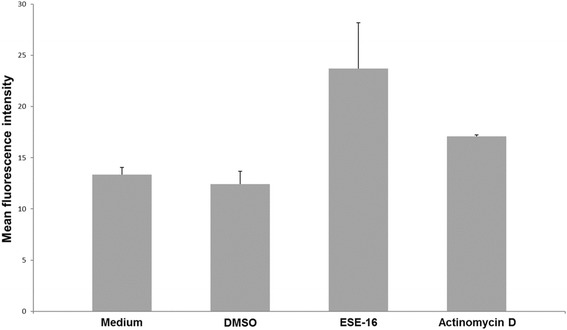
Bar graph of the average mean fluorescence intensity measured for the presence of early apoptosis in HeLa cells after exposure to ESE-16. The compound-exposed cells had an insignificant higher occurrence of early apoptosis than the control cells

Our data also revealed an average statistically insignificant increase in the MFI of the ESE-16-treated cells (23.7) compared to the controls (vehicle-treated control cells, which had an average of 12.4; Fig. 14). This statistically insignificant increase (*p* > 0.05) may be due to PS externalization being an early indicator of apoptosis. After 24 h of exposure, the cells may have already passed this early apoptotic phase. The average MFI for the positive control (actinomycin D-treated cells) was 17.75.

#### Apoptosis detection based on caspase 3

Caspase 3 is an executioner caspase known to be involved in the later degradation phase of apoptosis. To ascertain a further understanding of the signalling of ESE-16 and confirm apoptosis induction in the HeLa cells, caspase 3 detection utilizing a colorimetric assay was performed.

**Fig. 15:**
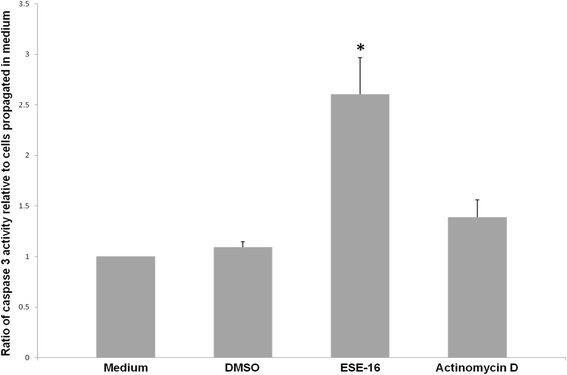
Bar graph illustrating the ratio of caspase 3 activity of vehicle-treated control, ESE-16-treated and actinomycin D-treated cells relative to cells propagated in medium after 24 h exposure. Compound-treated cells showed a statistically significant increase in the presence of caspase 3 compared to vehicle-treated control cells. *p < 0.05

The results revealed a statistically significant increase in caspase 3 activity following ESE-16 and actinomycin D exposure when compared to the vehicle-treated control cells (Fig. 15). HeLa cells exposed to ESE-16 showed an average-to-medium ratio of 2.6034, whereas the vehicle-treated control cells had a value of 1.0917, indicating that HeLa cells are in late phase apoptosis after 24 h of exposure to ESE-16.

## Discussion

One of the most successful groups of chemotherapeutics in the fight against cancer are those targeting and disrupting the microtubule dynamics of cancer cells [16, 17, 34, 62]. One such microtubule-targeting agent (MTA), 2ME, has demonstrated antiproliferative action both in vitro and in vivo against a variety of cancers [18, 20, 29, 63–65]. Considering its limitations and the outcomes of previous researched 2ME analogs, novel sulphamoylated estradiol derivatives were in silico-designed at our laboratory and synthesized to be tested in vitro. The aim was to maintain antimitotic properties, while improving bioavailability and potency [30].

The main focuses in the design of ESE-16 were the capabilities to disrupt microtubule dynamics, thus interfering with cell division leading to eventual cell death, and to inhibit CAIX, which is overexpressed in most tumors, to decrease tumor progression [30, 66, 67]. Previous studies showed that ESE-16 has an affinity to bind to both CAII and CAXI, and demonstrates higher bioavailablity in vitro than 2ME [30, 42].

This study investigated the in vitro effects of ESE-16 on cell proliferation, morphology, cell cycle progression, and apoptosis induction in a cervical carcinoma.

Cell proliferation studies revealed a statistically significant antiproliferative effect on HeLa cells after 24 h. The exposure concentration of 0.2 μM was calculated as the GI_50_, inhibiting cell proliferation to 50%, and was used for all subsequent experimentation. This antiproliferative activity exerted by ESE-16 was confirmed using the xCELLigence RTCA system. Stander et al. [30] performed growth studies on various carcinoma cell types, which demonstrated that ESE-16 decreased cell proliferation in the nanomolar range in cervical, oesophageal (SNO) and estrogen receptor-positive (MCF-7) and -negative (MDA-MB-231) breast cancer cell lines. Subsequent research has also demonstrated that ESE-16 significantly reduced HeLa cell growth at similar nanomolar concentrations, following 24 h of exposure [30, 41, 68, 69]. Dose-dependent studies have revealed antiproliferative activity of other sulphamoylated estradiol analogs, namely 2-MeOE2*bis*MATE, 2-ethyl-3-*O*-sulphamoyl-estra-1,3,5(10),15-tetraen-17-ol (ESE-15-ol), (8R,13S,14S,17S)-2-ethyl-13-methyl-7,8,9,11,12,13,14,15,16,17-decahydro-6H-cyclopenta[a]phenanthrane-3,17-diyl bis(sulphamate) (EMBS) and 2-ethyl-17-(1′-methylene)estra-1,3,5(10)-trien-3-O-sulphamate (compound 10 or C10) at these nanomolar ranges in various cancer types [30, 69–72].

Qualitative data produced by means of PlasDIC, light and confocal microscopy provided information on the changes in cell and microtubule morphology of cervical carcinoma cells after exposure to ESE-16. The H&E staining technique revealed a decrease in cell density and the presence of cells in metaphase block with visible features of apoptosis, such as hypercondensed chromatin, membrane blebbing, shrunken cells and apoptotic bodies.

Mitotic indices further revealed that treatment with ESE-16 caused a significant increase in the quantity of cells in metaphase and cells with abnormal characteristics associated with apoptosis. These quantitative data support the observational information obtained with PlasDIC and light microscopy and are comparable with results obtained by Wolmarans et al. [40] and Vorster et al. [73], who showed a rise in metaphase and apoptotic cells in SNO and MCF-7 cell lines respectively exposed to ESE-16 and 2-MeOE2*bis*MATE for 24 h.

Studies previously performed on 2ME, the parental molecule of ESE-16, showed that 2ME interacts with the microtubules by binding to tubulin at the colchicine site, inhibiting polymerization, which results in cell cycle arrest and subsequent cell death [19, 22, 29]. Confocal microscopy demonstrated that ESE-16 interferes with normal microtubule function and causes tubulin disintegration in cervical carcinoma cells. Visagie et al. [69] performed a tubulin polymerization assay that supported the latter results, indicating that ESE-16 caused depolymerizing activity and exerted a more pronounced effect compared to other 2ME analogs. These data therefore show that the chemical changes made to 2ME did not alter its target-binding affinity for tubulin. ESE-16 also exerted a negative effect on tubulin causing microtubule abrogation in SNO and MDA-MB-231 cells [40, 68, 69]. Similarly, other sulphamoylated 2ME analogues had the same influence on tubulin in various cancer cell types [48, 70, 72, 74, 75].

Mitotic indices were confirmed with cell cycle progression investigations, which revealed a statistically significantly greater number of HeLa cells in sub-G_1_ and G_2_/M phases, suggesting the presence of apoptotic cells and cells enduring a mitotic (metaphase) block, respectively. This finding was supported by a rise in the number of apoptotic and metaphase cells in oesophageal and estrogen receptor-positive and -negative breast carcinoma cells after ESE-16 exposure [30, 39, 68].

Further cell cycle analysis revealed a statistically significant upregulation of cyclin B1 in the ESE-16-treated HeLa cells, which is consistent with research performed in our laboratory on ESE-16 and another 2ME sulphamoylated analog, 2-MeOE2*bis*MATE [41, 42, 73]. This finding supported results from the cell cycle analysis suggesting activation of the spindle assembly checkpoint. Stander et al. [42] also showed through global gene and protein expression studies that ESE-16 increased protein levels of cyclin B in the MDA-MB-231 cell line. 2ME, paclitaxel (another microtubule-targeting agent) and 2-MeOE2*bis*MATE induced an upregulation of cyclin B levels in prostate and estrogen-receptor positive breast cancer cells, with 2-MeOE2*bis*MATE causing the strongest induction of the cyclin [57, 76]. High levels of cyclin B inhibit cell cycle progression and apoptosis, and only once the levels of cyclin B decrease, can the cells undergo complete apoptosis. It has been reported that cyclin B degradation can vary between different cell lines [77]. Newman et al. [78] demonstrated that after 24 h, 500 nM of paclitaxel and 2-MeOE2*bis*MATE induced p53-mediated apoptosis, with cyclin B levels declining after 48 h.

Annexin V–FITC further confirmed induction of apoptosis by ESE-16. In the compound-treated cells, 2.6% and 8.82% of HeLa cells were respectively in early and late apoptosis. This statistically insignificant result could be due to the time of termination. The HeLa cells may already have been present in the latter phases of apoptosis. Those results are compatible with previous research from our laboratory, wherein the detection of PS was insignificant at an exposure concentration of 0.5 μM on HeLa cells and at 0.2 μM on SNO cells [40, 41]. However, 0.2 μM of ESE-16 caused a significant increase in PS externalization after 24 and 48 h treatment of MCF-7 and MDA-MB-231 cells [42, 71]. Although ESE-16 seems to have variable effects in terms of apoptotic induction in different cell types, the characteristics and ability of 2ME sulphamoylated analogs to induce apoptosis in a variety of cell lines can be considered as retained.

Our data also indicated the upregulation of caspase 3, which is an executioner caspase of apoptosis responsible for the execution of definitive apoptotic events, such as condensation of the nucleus, DNA degradation, membrane blebbing and breakdown of caspase substrates, all of which lead to cell death [60, 79]. This activation of caspase 3 indicates that ESE-16 induces apoptosis and does so in a caspase-dependent manner. Research performed at our laboratory showed that ESE-16 also upregulated caspase 6 and 8 in HeLa, MCF-7 and MDA-MB-231 cells, with caspase 7 also activated in MCF-7 cells [41, 68, 69]. Various researchers have reported that 2ME and 2-MeOE2*bis*MATE initiate both the intrinsic and extrinsic apoptosis pathways through the depolarization of the mitochondrial membrane potential and consequent release of cyt c or upregulation of DR5 [29, 57, 77, 80–82]. The activation of the former caspases supports the idea that ESE-16 may also induce apoptosis via the involvement of both the extrinsic and intrinsic apoptotic pathways. This notion is further supported by the activation of caspase 6 and 8 in HeLa cells, with reduced mitochondrial membrane potential, after exposure to the sulphamoylated analogs, ESE-15-ol and EMBS [69, 83].

Studies performed at our laboratory showed that ESE-16 triggers apoptotic cell death in HeLa, MCF-7 and MDA-MB-231 cells similarly to the parental compound, 2ME [41, 68, 69]. Simultaneous induction of apoptosis and autophagy by 2ME and its sulphamoylated analogs has also been reported in various cancer cell types [39, 50, 57, 68, 69, 71, 84].

## Conclusion

We investigated the effect of a novel in silico-designed compound, ESE-16, in a human epithelial cervical carcinoma (HeLa) cell line. The results revealed that 24 h exposure to 0.2 μM ESE-16 inhibited cell growth through cell arrest via disruption of the microtubule network leading to the induction of apoptosis. Our data also demonstrate that the compound is more effective than its parental 2ME, indicating antiproliferative properties in the nanomolar versus the micromolar range. Future research will involve ex vivo and in vivo studies of this potential anticancer agent with the aim of improving and/or developing novel chemotherapeutics against cervical cancer.

## Abbreviations

2ME: 2-Methoxyestradiol
ATCC: American Tissue Culture Collection
C10: 2-Ethyl-17-(1′-methylene)estra-1,3,5(10)-trien-3-O-sulphamate
CA: Carbonic anhydrase
CDK: Cyclin-dependent kinase
CI: Cell index
DAPI: 4′,6-Diamidino-2-phenylindole
DMEM: Dulbecco’s modified Eagle medium
DMSO: Dimethyl sulphoxide
EMBS: (8R,13S,14S,17S)-2-ethyl-13-methyl-7,8,9,11,12,13,14,15,16,17-decahydro-6H cyclopenta[a]phenanthrane-3,17-diyl-bis(sulphamate)
ESE-15-ol: 2-Ethyl-3-O-sulphamoyl-estra-1,3,5(10),15-tetraen-17-ol
ESE-16: 2-Ethyl-3-O-sulphamoyl-estra-1,3,5(10)16-tetraene
FACS: Fluorescence-activated cell sorting
FCS: Fetal calf serum
FITC: Fluorescein isothiocyanate
GI_50_: 50% Growth inhibition
H&E: Hematoxylin and eosin;
HeLa: Cervical carcinoma cell line
HPV: Human papillomavirus
MCF-7: Estrogen receptor-positive breast carcinoma cell line
MDA-MB-231: Estrogen receptor-negative breast carcinoma cell line
MFI: Mean fluorescence intensity
MTA: Microtubule-targeting agent
PBS: Phosphate buffered saline
PI: Propidium iodide
PlasDIC: Polarization-optical transmitted light differential interference contrast
RNA: Ribonucleic acid;
RTCA: Real-time cell analyser
SNO: Oesophageal carcinoma cell line

## References

[1] Ferlay J, Soerjomataram I, Dikshit R, Eser S, Mathers C, Rebelo M (2015). Cancer incidence and mortality worldwide: sources, methods and major patterns in GLOBOCAN 2012. Int J Cancer.

[2] Vizcaino AP, Moreno V, Bosch FX, Muñoz N, Barros-Dios XM, Borras J (2000). International trends in incidence of cervical cancer: II. Squamous-cell carcinoma. Int J Cancer.

[3] Tay SK (2012). Cervical cancer in the human papillomavirus vaccination era. Curr Opin Obstet Gynecol.

[4] Al-Mansour Z, Verschraegen C (2010). Locally advanced cervical cancer: what is the standard of care?. Curr Opin Oncol.

[5] De Vuyst H, Alemany L, Lacey C, Chibwesha CJ, Sahasrabuddhe V, Banura C (2013). The burden of human papillomavirus infections and related diseases in sub-saharan Africa. Vaccine.

[6] Ginsberg GM, Edejer TT, Lauer JA, Sepulveda C (2009). Screening, prevention and treatment of cervical cancer—a global and regional generalized cost-effectiveness analysis. Vaccine.

[7] Waggoner SE (2003). Cervical cancer. Lancet.

[8] Schmidt M, Bastians H (2007). Mitotic drug targets and the development of novel anti-mitotic anticancer drugs. Drug Resis Updat.

[9] Zhou J, Giannakakou P (2005). Targeting microtubules for cancer chemotherapy. Curr Med Chem Anticancer Agents.

[10] Weaver BA, Cleveland DW (2005). Decoding the links between mitosis, cancer, and chemotherapy: the mitotic checkpoint, adaptation, and cell death. Cancer Cell.

[11] Chan K, Koh CG, Li H (2012). Mitosis-targeted anti-cancer therapies: where they stand. Cell Death Dis.

[12] Garrett MD (2001). Cell cycle control and cancer. Curr Sci.

[13] Johnson DG, Walker LC (1999). Cyclins and cell cycle checkpoints. Annu Rev Pharmacol Toxicol.

[14] LaVallee TM, Burke PA, Swartz GM, Hamel E, Agoston GE, Shah J (2008). Significant antitumor activity in vivo following treatment with the microtubule agent ENMD-1198. Mol Cancer Ther.

[15] Jordan MA (2002). Mechanism of action of antitumor drugs that interact with microtubules and tubulin. Curr Med Chem Anticancer Agents.

[16] Pasquier E, Kavallaris M (2008). Microtubules: a dynamic target in cancer therapy. IUBMB Life.

[17] Shi J, Orth JD, Mitchison T (2008). Cell type variation in responses to antimitotic drugs that target microtubules and kinesin-5. Cancer Res.

[18] Schumacher G, Neuhaus P (2001). The physiological estrogen metabolite 2-methoxyestradiol reduces tumor growth and induces apoptosis in human solid tumors. J Cancer Res Clin Oncol.

[19] Kamath K, Okouneva T, Larson G, Panda D, Wilson L, Jordan MA (2006). 2-Methoxyestradiol suppresses microtubule dynamics and arrests mitosis without depolymerizing microtubules. Mol Cancer Ther.

[20] Li L, Bu S, Backstrom T, Landstrom M, Ulmsten U, Fu X (2004). Induction of apoptosis and G2/M arrest by 2-methoxyestradiol in human cervical cancer HeLaS3 cells. Anticancer Res.

[21] Mooberry SL (2003). Mechanism of action of 2-methoxyestradiol: new developments. Drug Resis Updat.

[22] Lakhani NJ, Sarkar MA, Venitz J, Figg WD (2003). 2-Methoxyestradiol, a promising anticancer agent. Pharmacotherapy.

[23] Lakhani NJ, Sparreboom A, Xu X, Veenstra TD, Venitz J, Dahut WL (2007). Characterization of in vitro and in vivo metabolic pathways of the investigational anticancer agent, 2-methoxyestradiol. J Pharm Sci.

[24] Risinger AL, Giles FJ, Mooberry SL (2009). Microtubule dynamics as a target in oncology. Cancer Treat Rev.

[25] Matei D, Schilder J, Sutton G, Perkins S, Breen T, Quon C (2009). Activity of 2 methoxyestradiol (Panzem NCD) in advanced, platinum-resistant ovarian cancer and primary peritoneal carcinomatosis: a Hoosier oncology group trial. Gynecol Oncol.

[26] Tevaarwerk A, Holen K, Alberti D, Sidor C, Arnott J, Quon C (2009). Phase I trial of 2-methoxyestradiol nanocrystal dispersion in advanced solid malignancies. Clin Cancer Res.

[27] Harrison MR, Hahn NM, Pili R, Oh WK, Hammers H, Sweeney C (2011). A phase II study of 2-methoxyestradiol (2ME2) NanoCrystal® dispersion (NCD) in patients with taxane-refractory, metastatic castrate-resistant prostate cancer (CRPC). Investig New Drugs.

[28] Bruce JY, Eickhoff J, Pili R, Logan T, Carducci M, Arnott J (2012). A phase II study of 2-methoxyestradiol nanocrystal colloidal dispersion alone and in combination with sunitinib malate in patients with metastatic renal cell carcinoma progressing on sunitinib malate. Investig New Drugs.

[29] Mueck A, Seeger H (2010). 2-Methoxyestradiol—biology and mechanism of action. Steroids.

[30] Stander A, Joubert F, Joubert A (2011). Docking, synthesis, and in vitro evaluation of antimitotic estrone analogs. Chem Biol Drug Des.

[31] Stander XX, Stander BA, Joubert AM (2011). In vitro effects of an in silico-modelled 17-estradiol derivative in combination with dichloroacetic acid on MCF-7 and MCF-12A cells. Cell Prolif.

[32] Chander SK, Foster PA, Leese MP, Newman SP, Potter BV, Purohit A (2007). In vivo inhibition of angiogenesis by sulphamoylated derivatives of 2-methoxyoestradiol. Br J Cancer.

[33] Visagie M, Mqoco T, Joubert A (2012). Sulphamoylated estradiol analogue induces antiproliferative activity and apoptosis in breast cell lines. Cell Mol Biol Lett.

[34] Pasquier E, Sinnappan S, Munoz MA, Kavallaris M (2010). ENMD-1198, a new analogue of 2-methoxyestradiol, displays both antiangiogenic and vascular-disrupting properties. Mol Cancer Ther.

[35] Visagie MH, Joubert AM (2010). The in vitro effects of 2-methoxyestradiol-bis-sulphamate on cell numbers, membrane integrity and cell morphology, and the possible induction of apoptosis and autophagy in a non-tumorigenic breast epithelial cell line. Cell Mol Biol Lett.

[36] Ho Y, Purohit A, Vicker N, Newman S, Robinson J, Leese M (2003). Inhibition of carbonic anhydrase II by steroidal and non-steroidal sulphamates. Biochem Biophys Res Commun.

[37] Ireson C, Chander S, Purohit A, Perera S, Newman S, Parish D (2004). Pharmacokinetics and efficacy of 2-methoxyoestradiol and 2-methoxyoestradiol-bis-sulphamate in vivo in rodents. Br J Cancer.

[38] Leese MP, Leblond B, Newman SP, Purohit A, Reed MJ, Potter BV (2005). Anti-cancer activities of novel D-ring modified 2-substituted estrogen-3-O-sulfamates. J Steroid Biochem Mol Biol.

[39] Wolmarans E, Mqoco T, Stander A, Nkandeu S, Sippel K, McKenna R (2014). Novel estradiol analogue induces apoptosis and autophagy in esophageal carcinoma cells. Cell Mol Biol Lett.

[40] Wolmarans E, Sippel K, McKenna R, Joubert A (2014). Induction of the intrinsic apoptotic pathway via a new antimitotic agent in an esophageal carcinoma cell line. Cell Biosci.

[41] Theron AE, Nolte EM, Lafanechere L, Joubert AM (2013). Molecular crosstalk between apoptosis and autophagy induced by a novel 2-methoxyestradiol analogue in cervical adenocarcinoma cells. Cancer Cell Int.

[42] Stander BA, Joubert F, Tu C, Sippel KH, McKenna R, Joubert AM (2013). Signaling pathways of ESE-16, an antimitotic and anticarbonic anhydrase estradiol analog, in breast cancer cells. PLoS One.

[43] Gillies RJ, Didier N, Denton M (1986). Determination of cell number in monolayer cultures. Anal Biochem.

[44] Kueng W, Silber E, Eppenberger U (1989). Quantification of cells cultured on 96-well plates. Anal Biochem.

[45] Abassi YA, Xi B, Zhang W, Ye P, Kirstein SL, Gaylord MR (2009). Kinetic cell-based morphological screening: prediction of mechanism of compound action and off-target effects. Chem Biol.

[46] Atienzar FA, Tilmant K, Gerets HH, Toussaint G, Speeckaert S, Hanon E (2011). The use of real-time cell analyzer technology in drug discovery: defining optimal cell culture conditions and assay reproducibility with different adherent cellular models. J Biomol Screen.

[47] Kirstein SL, Atienza JM, Xi B, Zhu J, Yu N, Wang X (2006). Live cell quality control and utility of real-time cell electronic sensing for assay development. Assay Drug Dev Technol.

[48] Marais S, Mqoco T, Stander A, Van Papendorp D, Joubert A (2012). The in vitro effects of a sulphamoylated derivative of 2-methoxyestradiol on cell number, morphology and alpha-tubulin disruption in cervical adenocarcinoma (HeLa) cells. Biomed Res.

[49] Wehner E (2003). PlasDIC, an innovative relief contrast for routine observation in cell biology. Imaging Microsc.

[50] Mqoco T, Joubert A (2012). 2-Methoxyestradiol-bis-sulphamate induces apoptosis and autophagy in an oesophageal carcinoma (SNO) cell line. Biomed Res-India.

[51] Avwioro G (2011). Histochemical uses of haematoxylin—a review. JPCS.

[52] Picot J, Guerin CL, Le Van Kim C, Boulanger CM (2012). Flow cytometry: retrospective, fundamentals and recent instrumentation. Cytotechnology.

[53] Darzynkiewicz Z, Halicka HD, Zhao H (2010). Analysis of cellular DNA content by flow and laser scanning cytometry. Adv Exp Med Biol.

[54] Pozarowski P, Darzynkiewicz Z (2004). Analysis of cell cycle by flow cytometry. Methods Mol Biol.

[55] Mollinedo F, Gajate C (2003). Microtubules, microtubule-interfering agents and apoptosis. Apoptosis.

[56] Domingo-Sananes MR, Kapuy O, Hunt T, Novak B (2011). Switches and latches: a biochemical tug-of-war between the kinases and phosphatases that control mitosis. Proc R Soc Lond B Biol Sci.

[57] Stander BA, Marais S, Vorster C, Joubert AM (2010). In vitro effects of 2-methoxyestradiol on morphology, cell cycle progression, cell death and gene expression changes in the tumorigenic MCF-7 breast epithelial cell line. J Steroid Biochem Mol Biol.

[58] Vermes I, Haanen C, Steffens-Nakken H, Reutellingsperger C (1995). A novel assay for apoptosis flow cytometric detection of phosphatidylserine expression on early apoptotic cells using fluorescein labelled annexin V. J Immunol Methods.

[59] van Engeland M, Nieland LJ, Ramaekers FC, Schutte B, Reutelingsperger CP (1998). Annexin V-affinity assay: a review on an apoptosis detection system based on phosphatidylserine exposure. Cytometry.

[60] Fan TJ, Han LH, Cong RS, Liang J (2005). Caspase family proteases and apoptosis. Acta Biochim Biophys Sin.

[61] Mirnikjoo B, Balasubramanian K, Schroit AJ (2009). Suicidal membrane repair regulates phosphatidylserine externalization during apoptosis. J Biol Chem.

[62] Peyrat J, Brion J, Alami M (2012). Synthetic 2-methoxyestradiol derivatives: structure-activity relationships. Curr Med Chem.

[63] Choi HJ, Zhu BT (2012). Critical role of cyclin B1/Cdc2 up-regulation in the induction of mitotic prometaphase arrest in human breast cancer cells treated with 2-methoxyestradiol. Biochim Biophys Acta.

[64] Chua YS, Chua YL, Hagen T (2010). Structure activity analysis of 2-methoxyestradiol analogues reveals targeting of microtubules as the major mechanism of antiproliferative and proapoptotic activity. Mol Cancer Ther.

[65] LaVallee TM, Zhan XH, Herbstritt CJ, Kough EC, Green SJ, Pribluda VS (2002). 2-Methoxyestradiol inhibits proliferation and induces apoptosis independently of estrogen receptors alpha and beta. Cancer Res.

[66] van Vuuren RJ, Visagie MH, Theron AE, Joubert AM (2015). Antimitotic drugs in the treatment of cancer. Cancer Chemother Pharmacol.

[67] Thiry A, Dogne J, Masereel B, Supuran CT (2006). Targeting tumor-associated carbonic anhydrase IX in cancer therapy. Trends Pharmacol Sci.

[68] Nkandeu DS, Mqoco TV, Visagie MH, Stander BA, Wolmarans E, Cronje MJ (2013). In vitro changes in mitochondrial potential, aggresome formation and caspase activity by a novel 17-β-estradiol analogue in breast adenocarcinoma cells. Cell Biochem Funct.

[69] Visagie M, Theron A, Mqoco T, Vieira W, Prudent R, Martinez A (2013). Sulphamoylated 2-methoxyestradiol analogues induce apoptosis in adenocarcinoma cell lines. PLoS One.

[70] Stander BA, Joubert F, Tu C, Sippel KH, McKenna R, Joubert AM (2012). In vitro evaluation of ESE-15-ol, an estradiol analogue with nanomolar antimitotic and carbonic anhydrase inhibitory activity. PLoS One.

[71] Visagie MH, Stander BA, Birkholtz L, Margaretha A (2013). Short communication: effects of a 17-beta estradiol analogue on gene expression and morphology in a breast epithelial adenocarcinoma cell line: a potential antiproliferative agent. Biomed Res.

[72] Leese MP, Leblond B, Smith A, Newman SP, Di Fiore A, De Simone G (2006). 2-substituted estradiol bis-sulfamates, multitargeted antitumor agents: synthesis, in vitro SAR, protein crystallography, and in vivo activity. J Med Chem.

[73] Vorster C, Joubert A (2010). In vitro effects of 2-methoxyestradiol-bis-sulphamate on cell growth, morphology and cell cycle dynamics in the MCF-7 breast adenocarcinoma cell line. Biocell.

[74] Mqoco T, Marais S, Joubert A (2010). Influence of estradiol analogue on cell growth, morphology and death in esophageal carcinoma cells. Biocell.

[75] Theron A, Prudent R, Nolte E, van den Bout I, Punchoo R, Marais S (2015). Novel in silico-designed estradiol analogues are cytotoxic to a multidrug-resistant cell line at nanomolar concentrations. Cancer Chemother Pharmacol.

[76] Newman SP, Ireson CR, Tutill HJ, Day JM, Parsons MF, Leese MP (2006). The role of 17beta-hydroxysteroid dehydrogenases in modulating the activity of 2-methoxyestradiol in breast cancer cells. Cancer Res.

[77] Ho YT, Foster PA, Newman SP, Leese MP, Potter BV, Purohit A (2006). Sulphamoylated derivatives of 2-methoxyestradiol induce apoptosis in breast, ovarian and prostate cancer cell lines through mitotic arrest via the intrinsic apoptotic pathway. Cancer Res.

[78] Newman SP, Foster PA, Ho YT, Day JM, Raobaikady B, Kasprzyk PG (2007). The therapeutic potential of a series of orally bioavailable anti-angiogenic microtubule disruptors as therapy for hormone-independent prostate and breast cancers. Br J Cancer.

[79] Slee EA, Adrain C, Martin SJ (2001). Executioner caspase-3, −6, and −7 perform distinct, non-redundant roles during the demolition phase of apoptosis. J Biol Chem.

[80] LaVallee TM, Zhan XH, Johnson MS, Herbstritt CJ, Swartz G, Williams MS (2003). 2-Methoxyestradiol up-regulates death receptor 5 and induces apoptosis through activation of the extrinsic pathway. Cancer Res.

[81] Foster PA, Ho YT, Newman SP, Kasprzyk PG, Leese MP, Potter BV (2008). 2-MeOE2bisMATE and 2-EtE2bisMATE induce cell cycle arrest and apoptosis in breast cancer xenografts as shown by a novel ex vivo technique. Breast Cancer Res Treat.

[82] Kato S, Sadarangani A, Lange S, Delpiano AM, Vargas M, Branes J (2008). 2-Methoxyestradiol mediates apoptosis through caspase-dependent and independent mechanisms in ovarian Cancer cells but not in normal counterparts. Reprod Sci.

[83] Visagie MH, Birkholtz LM, Joubert AM (2015). A 2-methoxyestradiol bis-sulphamoylated derivative induces apoptosis in breast cell lines. Cell Biosci.

[84] Chen Y, McMillan-Ward E, Kong J, Israels S, Gibson S (2008). Oxidative stress induces autophagic cell death independent of apoptosis in transformed and cancer cells. Cell Death Differ.

